# Concerning the Causes of Faillures in the Use of Cohesive Gold Foil; the Mallet, Etc.

**Published:** 1877-05

**Authors:** H. L. Sage


### ARTICLE V.

### *Concerning the Causes of Failures in the Use of Cohesive Foil, the Mallet, Etc.*

BY H. L. SAGE, D. D. S.

Thoroughness is, doubtless, an element of success which does not enter into the practice of one-half of those who constitute the numerical force of the dental profession. Is not this apparent to any one who has himself the grace, the ability, and the determination to utilize such an element, and to judge by observation and experience of the character of an operation?

By success, is not meant *pecuniary plethora*, but that which compasses the ends which should be sought—the best possible results which are attainable by conservative treatment, considered with regard to dental practice.

Success, then, being contingent on such an issue, its attainment appears to be the exception and not the rule, with very many.

In this connection, reference will be made to one of the directions in which failures are said to be so common,—the use of cohesive foil in operative dentistry,—because, as some maintain, we have gone to extremes in its employment, and are not eclectic enough in our practice as regards the blending of old and well tried methods, with those of more recent date.

As to failures connected with the use of cohesive foil it may be well to canvass the subject sufficiently to determine whether the fault is in the material itself, or the manner of its employment.

Whether better operations every way, may not be made with this preparation of gold than with any other, is hardly a question worthy of discussion, when considered with regard to beauty of finish, durability, and the effect to check the progress of dental caries, and this, aside from the influences attended upon defectively organized tooth structure, and constitutional tendencies, which in themselves, predispose to, or invite an attack of caries ; so that here, mere mechanical manipulation will be considered, both with regard to the preparation of the cavity and for the reception of the gold, and the manner of its introduction, prophylactic treatment and constitutional effects, so far even, as they have a bearing upon the success of an operation, not entering into consideration in this paper.

Some of our confreres are assuming that we should return to first principles; to the old fashioned methods of operating; that what we have been wont to consider improvements in practice have proved unsuccessful retrograde movements, many of them at least ; and that among these are the excessive use of cohesive foil and mallet force, the too frequent restoration of contour, the abandonment of permanent separations, etc.

There may have been some force in these assumptions, as related to the employment of heavy foil, and the almost total abandonment of V shaped spaces in crowded teeth.

That in many cases, permanent separations are desirable is doubtless true, but that in most it is not advisable to make them is also true.

*Some*, the writer among the number, are inclined to think that careless and imperfect operators are responsible for most of the failures, leaving out of view defectively constituted teeth, and local or chemical action.

It is probably safe to assert, that more teeth, in proportion to the number filled, have been saved by the use of cohesive foil and mallet force, during the time they have been in vogue, than in the same length of time previously by the employment of soft foil and the old fashioned methods of operating; and thousands of teeth are now saved and salvable, that could not have been preserved, and are not capable of being by other means.

Then again, great improvements in the preparation of cavities have been made; more thorough following up and cutting out of interstitial decay, the blending of contiguous cavities, etc., have been practiced. So far as filling is concerned, more perfect results can be attained with foil which is cohesive than with other preparations of gold, and that too in every case. But great care must be exercised.

It may be that less care, certainly less time and material is required in the use of soft foil, than is requisite when the cohesive is employed. But that is no argument in favor of the former, unless as valuable results can be had with it as with the latter.

Do perfectly moisture-tight fillings of non cohesive foil often greet our vision? No.

Witness the discoloration about the labial and palatal surfaces of many teeth in which soft foil fillings are remaining, produced by the ingress of fluids about the borders of cavities, and in which decay is going on under the gold. When cohesive foil is used, this is entirely unnecessary—always in the case of the front teeth inexcusable, though caries may work up to and undermine a properly inserted filling.

Do or can soft or non-adhesive foil fillings, (using the terms synonomously,) be made to present that polished and highly finished surface so pleasing to the eye, so cleanly, and so capable of preventing or repelling the lodgment of foreign matters, which renders well condensed fillings of cohesive foil superior to all others? No.

Are not hard, well finished and polished surfaces required in order to permit of perfect cleanliness about the borders of the filling? They are.

Can they be made free from indentations, roughness and porosity with soft foil? Hardly.

Is it not essential that a filling should present as smooth a surface, and be as highly polished as the enamel, in order to obtain the best results? It is.

Is it not a very difficult matter to produce such a surface with soft foil? It is.

By soft foil is meant that which has not been made adhesive by subjecting it to the action of heat.

Will soft foil fillings wear well when subjected to much friction? No.

Can contour fillings be made of soft foil? No—and semi-adhesive is not entirely reliable for such work.

Because the borders of a cavity have been broken down, nicked, disintegrated or roughened by the employment of too much force in malleting, that is, by carelessness, is the abuse of a good thing an argument against its use? Many a border has been thus fractured, and ingress thus afforded for agents productive of decay, at the cervical border especially.

Extreme care in packing and condensing is always necessary to prevent failures at this point. And the same may be said, with less stress perhaps, of the labial or buccal and palatal or lingual borders.

A bicuspid tooth, with a cavity involving the proximal and grinding surface, and extending to or under the margin of the gum, so as to leave at the cervical border a narrow neck of enamel, is a case in point.

A little digression, a word, as to the rubber dam. It is well before preparing the cavity to adjust this appliance.

The advantages are obvious. A better view of the cavity is obtained, and the relative proximity of caries to the pulp more easily noted. Time is saved; one is not under the necessity of mopping out the saliva, or removing the detached debris by the slow and somewhat painful process of hoeing it out, for the chips or powdered dentine can be instantly removed by one puff with the air syringe.

The cavity being kept perfectly dry, the sensitiveness of the dentine attendant upon the removal of the decayed portion is, by the absence of moisture, very much lessened; and lastly, when the gold is introduced, you need not labor under the constant terror and disadvantage of being threatened or overwhelmed by the fluids of the mouth, and you will be much more likely to produce a fine operation, by means of more thorough and deliberate packing and perfect welding. Of other advantages pertaining thereto, it is not necessary to speak.

A word as to wedging.

Gradual wedging is preferable in most cases, for the obtainment of room, if sufficient space is not already gained. It is less injurious to the tissues than rapid wedging, and most patients can better bear moderate pain longer continued than that which is extreme, though lasting but a moment, and will give you the credit of being less barbarous, if you adopt the former method in preference to the latter; a result borne out by observation and tending much to enhance the personal popularity of the dentist, as the avoidance of all unnecessary pain will naturally do.

After obtaining the requisite amount of space, and subsebuent to adjusting the dam, a wedge may be inserted with the fingers at the base of the cavity; but in no instance should it be allowed to overlap the latter—a disadvantage that sometimes pertains to rapid wedging, for the reason that then, the wedge is indispensable during the operation, even though the cavity may extend under the margin of the gum. Though a wedge here is useful not only for the purpose of steadying the tooth to be filled, maintaining the space already gained, preventing injury to the dam by the burs of the engine, and as a protection to the cervical border while malleting and introducing the filling, yet it is often the case that the caries extends so far that the wedge can not be inserted without covering a portion of the cavity, or unduly lacerating the gum and causing unnecessary suffering; it is then productive of more harm than good for obvious reasons, and to be avoided if possible; besides the operation can be made perfect if the wedge is dispensed with,—an advantage connected with gradual wedging.

When the borders of a cavity are fragile or jagged, they should be cut or trimmed until firm or at least well defined margins are secured; for when they are very thin, it is not always possible to obtain sufficient strength, especially in the grinding teeth, without cutting away extensively and restoring with gold; and in such cases, the pulp is often nearly or quite exposed, so that treatment has to be instituted by capping or otherwise, preliminary to the insertion of the filling.

But to simplify. If the tooth is a bicuspid, let the cavity under consideration involve the proximal surface, with the coronal floor broken down or so thin as to render it liable to fracture in mastication. In the latter case it is much better to cut it away; for a grinding surface of welded gold is much preferable to one composed of an attenuated floor or roof of tooth structure, while its removal renders the operation of filling with adhesive foil less difficult, and success more certain, so far as conservation of the tooth is concerned; but when non-adhesive foil is inserted, it is more necessary that this weak grinding surface should be retained, if it still remains, in order to secure the filling and prevent it from wearing away by the friction attendant upon mastication.

After removing the carious and friable portions, if retaining points are made use of, (these or grooves seeming to be necessary for anchorage) two, at the bottom of the cavity, near the junction of the dentine and enamel, will suffice—

one pointing towards the lingual and the other in the direction of the buccal surface, with a third larger one in the back portion of the cavity near the grinding, and pointing obliquely upward or downward, according as the tooth is an inferior or superior one. To these may be added, when the walls are of sufficient thickness to permit it, a narrow groove or under cut near the buccal and lingual border, and extending a short distance from the cervical portion. With these precautions, dove-tailing on the grinding surface is unnecessary, and would cause weakening of the tooth, without any compensating advantage.

Now if the foil is not made harsh by over heating, adhesive or perhaps more properly cohesive as regards terms, is the best that can be employed for filling the retaining points and building up to and overlapping the delicate cervical border of the cavity, and that too, by the use of mallet force, and by careful packing.

Pluggers with small points, small, loosely rolled pellets and gentle malleting will enable one to perfectly cover the borders of the cavity, solidly, without leakage, fracture or pulvurent tooth substance.

But this cervical border should present a flat or level surface, that is, on the short diameter, with more or less concavity at the buccal and palatine angles; not a sharp ragged edge liabel to break down or cut the gold, while applying the force necessary to condense it.

If ropes are used, they should be small and loosely rolled (with a napkin) especially for adaptation to the bottom and sides of the cavity. They may be annealed and cut into pellets of various lengths, according to the requirements of the case.

It is quite superfluous to cut before annealing, and then pass the gold through the flame of the lamp pellet by pellet. It requires more time than if the rolls of foil are annealed first and cut afterward, and they will work nicely without this extra trouble, if the ropes are not made too hard by over heating and rolling too tightly. When large pellets are used, of course time is not so much of an item. But most operators use too large masses of gold, and pluggers with too large condensing surfaces about the borders of cavities. Herein lies one cause of failure.

Small pellets, small plugger points, and light blows with the mallet, are very much the best for packing and condensing the gold about the walls of a cavity, and will make a perfect operation, with no crumbling, fractured walls; and this whether ropes cut into pellets, or ribbons are used, the sides of the filling meanwhile being kept a trifle higher than the central portions. For filling about the walls of ordinary cavities, a sheet of number four should make at least five or six full length, loosely rolled ropes, to be cut into pellets.

Large pellets or full length ropes, not too tightly rolled, may be worked into the central portions of the filling, with pluggers presenting larger serrated surfaces, and by more pronounced mallet force.

If this method of operating is carried out until the filling is completed, there need be no failures with cohesive foil, so far as success depends on the operation itself.

To repeat, the majority of operators are in the habit of trying to condense too large masses of foil, use pluggers with too large condensing surfaces in contiguity to the walls of cavities and exercise too little care in adapting the gold thereto so that not only more force is required to weld it, but the danger of disintegrating the walls is much increased.

Besides caverns or spaces are often left about the borders, or contiguous to the walls of the cavity, owing to imperfect packing and welding, and choking up of the material from the before mentioned causes.

It is not surprising that so many failures occur to one who views the careless and hurried manner in which cohesive foil is often manipulated, even by many who claim to be first and stand high as operators.

*Careless* D. D. S.'s, and in exceptional cases others, may be able to manipulate soft foil to the best advantage. Very careful ones should be competent to attain the best results with cohesive foil.

There is one other point to which attention may be briefly directed.

Some operators are in the habit of packing the inner portions of a filling quite loosely, even about the walls of a cavity.

They think perhaps, that if the gold is well condensed near the surface of a filling, and in the retaining points, it is quite unnecessary to be particular in other respects.

Take, for example, a large grinding surface cavity in a molar, with a well condensed and moisture tight gold filling so far as the outer surface is concerned, but with the inner portions imperfectly adapted to the walls of the cavity, and this, whether non-adhesive or adhesive foil is employed; for some commence with one and finish off with the other.

Such a grinding surface filling may do good service for a time, though it certainly does not represent a fine operation through and through, nor, in many cases, a safe one.

If the tooth subsequently decays on the proximal surface, and is not seasonably filled, the caries may extend under and about the filling in the grinding surface, so as to endanger the pulp and cause discoloration of the tooth, if the original cavity was a large one, and thus necessitate the removal of operator number one's filling in order to make a safe thing of it.

Here is an under cut by caries, facilitated by a substratum of soft, porous, spongy and loosely packed gold and preceded in the first instance, it may be, by an under cut in prices. Have not such cases fallen under the observation of most?

Do you wonder that A can operate for less money than B, and that his patients think him very reasonable in his charges?

The mystery is solved! A's filling is hard without, and very, very soft within. He charges less and saves his time and strength, but slights the operation in order to do it.

He makes more money, perhaps gains more patients than B, because he acts on the principle that an under cut in price may be easily balanced by an under cut in quality, provided the defect don't appear on the face of it until a reasonable length of time has elapsed.

But if in a similar case, the main portion of a filling is composed of oxy-chloride, in the event of decay appearing on the proximal surface, the same care would need to be exercised, lest the oxy-chloride becomes exposed, and softened by the fluids of the mouth.

This affords an objection to filling a considerable portion of a cavity with this material. But of course this is necessary where it is used as a capping.

But these observations will suffice, though presenting but a partial and imperfect view of the points under consideration.—*Dental Register.*

---

